# The Effectiveness of a Topical Turmeric-Based Formulation on Contact Dermatitis: A Randomized, Double-Blind, Controlled Trial

**DOI:** 10.5812/ijpr-172121

**Published:** 2026-08-18

**Authors:** Shabnam Khatami, Seyede Nargess Sadati Lamardi, Roya Najafi-Vosough, Azadeh Mizani, Laila Shirbeigi, Alireza Abbassian

**Affiliations:** 1 Department of Traditional Medicine, School of Persian Medicine, Tehran University of Medical Sciences, Tehran, Iran; 2 Traditional Persian Medicine and Complementary Medicine (PerCoMed) Student Association, Students' Scientific Research Center, Tehran University of Medical Sciences, Tehran, Iran.; 3 Department of Traditional Pharmacy, School of Persian Medicine, Tehran University of Medical Sciences, Tehran, Iran; 4 Research Center for Health Sciences, Institute of Health Sciences and Technology, Hamadan University of Medical Sciences, Hamadan, Iran; 5 Department of Parasitology, Pasteur Institute of Iran, Tehran, Iran

**Keywords:** Allergic Contact Dermatitis, *Aristolochia rotunda*, *Lawsonia inermis*, *Curcuma longa* Extract, Persian Medicine, Topical Herbal Drugs

## Abstract

**Background:**

Contact dermatitis (CD) affects approximately 15% of adults worldwide, and commonly used treatments are often associated with adverse effects. Traditional medicine sources, including Persian medicine, offer promising alternatives. Based on the principles of Persian medicine, the formulation investigated in this study combines *Curcuma longa* L., *Lawsonia inermis* L., and *Aristolochia rotunda* L. (CLA) to achieve a synergistic therapeutic effect.

**Objectives:**

This study aimed to evaluate the efficacy and safety of a 4-week intervention using CLA cream in patients with mild-to-moderate CD.

**Methods:**

A randomized, double-blind, placebo-controlled trial was conducted in 64 participants who were assigned to apply either CLA cream or the base cream. Assessments included demographic characteristics, the Dermatology Life Quality Index (DLQI), the Three-Item Severity Score (TIS), and skin parameters, including transepidermal water loss (TEWL), stratum corneum hydration, skin pH, and sebum production. Statistical analyses were performed using repeated-measures analysis of variance with least significant difference post hoc tests, Friedman tests, and Wilcoxon tests.

**Results:**

The CLA group showed significantly greater decreases in TEWL and TIS scores than the control group (P = 0.004 and P = 0.003, respectively). In the within-group analysis, sebum production increased significantly after the intervention in the CLA group (P = 0.006). *Stratum corneum* hydration improved significantly in both groups.

**Conclusions:**

A 4-week course of CLA cream appears to ameliorate inflammatory symptoms in patients with mild-to-moderate contact dermatitis. Further large-scale, longer-duration trials are warranted to establish the clinical utility of CLA cream as a complementary therapeutic option.

## 1. Background

Contact dermatitis (CD) is a prevalent inflammatory disorder affecting approximately 15% of the global adult population (1). It develops when the skin becomes irritated or inflamed after exposure to a substance that triggers an allergic reaction. Clinical symptoms vary according to disease severity and duration. CD is common among individuals whose occupations involve frequent chemical exposure. Regular handwashing with detergents or disinfectants, recommended as a preventive measure during the COVID-19 pandemic, also contributed to an increased incidence of dermatitis (2).

Conventional treatments for CD include nonpharmacological measures, topical medications, oral medications, and phototherapy. Topical corticosteroids are commonly used to treat CD; however, prolonged use of high-potency corticosteroids over large skin areas may cause adverse effects such as adrenal suppression, skin thinning, telangiectasia, folliculitis, and CD. Other treatments may weaken the immune system, and phototherapy may also cause adverse effects (3, 4). Given the costly process of accidental drug discovery (5), complementary and alternative medicine (CAM) may provide promising therapeutic sources. The growing popularity of complementary treatments further supports investigation in this field.

Persian medicine (PM), with historical origins spanning approximately 10,000 years, is a CAM system encompassing knowledge used for the diagnosis, prevention, and treatment of diseases in ancient Persia and transmitted across generations (6). PM offers various formulations for the treatment of CD. One formulation investigated in this study, termed CLA, consists of *Curcuma longa* L. (turmeric), *Lawsonia inermis* L. (henna), and *Aristolochia rotunda* L. (birthwort). Evidence suggests that these herbs have anti-inflammatory, antimicrobial, tissue-repair, and anticancer properties (7-9). Their accessibility in Iranian and Asian herbal markets, together with these properties, makes investigation of the CLA formulation practical and scientifically relevant.

## 2. Objectives

The aim of the present study was to evaluate the efficacy, anti-inflammatory potential, and biophysical effects on the skin of a CLA formulation consisting of *Curcuma longa*, *Lawsonia inermis*, and *Aristolochia rotunda*, compared with a control cream, in patients with mild-to-moderate contact dermatitis over 4 weeks. The Dermatology Life Quality Index (DLQI) was also assessed, and the safety and potential adverse effects of the formulation were monitored throughout the trial.

## 3. Methods

### 3.1. Study Design

Adult volunteers were recruited from the Center for Research and Training in Skin Diseases and Leprosy, Tehran, Iran, from March 2023 to March 2024. A dermatologist clinically confirmed the diagnosis, after which eligible participants were randomly allocated to the CLA or control group. Participants completed a demographic form and provided written informed consent. This trial was registered with the Iranian Registry of Clinical Trials (IRCT20220321054336N1). The protocol was approved by the Research Ethics Committee of Tehran University of Medical Sciences, Tehran, Iran (No. IR.TUMS.MEDICINE.REC.1400.1533).

### 3.2. Plant Material and Extraction

*Curcuma longa* L., *Lawsonia inermis* L., and *Aristolochia rotunda* L. were purchased from an herbal market in Tehran, Iran. The plant materials were authenticated by the Herbarium of the Faculty of Pharmacy, Tehran University of Medical Sciences, with voucher numbers PMP-459, PMP-1233, and PMP-1232, respectively.

*C. longa* rhizomes were crushed and macerated in 70% ethanol at room temperature for 24 hours; this process was repeated twice. The extract was then filtered and concentrated. *L. inermis* leaves were powdered. One hundred grams of powder was added to 1000 mL of distilled water, boiled for 10 minutes, and filtered. The extract was concentrated using a vacuum rotary evaporator. *A. rotunda* rhizomes were extracted by macerating the powder in distilled water for 24 hours. The mixture was filtered and concentrated. Each extract was dried in a vacuum oven at 40 °C.

### 3.3. CLA and Control Cream Preparation and Standardization

The CLA cream was prepared by combining *C. longa*, *L. inermis*, and *A. rotunda* extracts in a 3:2:1 ratio. This mixture was incorporated into a cream base (Farabi Base® cream, Iran) to achieve a final extract concentration of 4%. The specified ratio was derived from Persian medicine sources. The control cream was prepared by mixing the cream base with a pharmaceutical colorant (Sunset Yellow, Khat Zard®, Iran). Both creams were packaged in identical tubes.

The CLA formulation was standardized by quantitative analysis of marker compounds. Total phenolic content was determined using the Folin-Ciocalteu reagent (FCR) method (10) and calculated from the regression equation of a gallic acid calibration curve. Results were expressed as gallic acid equivalents (GAE; Sigma-Aldrich). Absorbance for the total phenol assay was measured at 765 nm.

Total phenols were measured in the cream samples. For extraction, 20 mL of boiling water was added to 2 g of cream. The mixture was stirred using a magnetic stirrer for 30 minutes and then filtered. The resulting filtrate was used to determine total phenolic content.

Given the documented toxicity of aristolochic acids in *A. rotunda*, quantification of their concentration in the extract was essential to ensure that it remained below toxic levels. The analysis was performed using high-performance liquid chromatography (HPLC), and test and standard solutions were prepared according to a validated method by Chang et al. (11).

The analysis was conducted using an HPLC system (Knauer Azura, Germany) equipped with a diode-array detector and an Eclipse XDB-C18 analytical column (15 cm × 4.6 mm, 5 μm). The mobile phase consisted of acetonitrile:water (45:55) with 2.0% acetic acid. The flow rate was 1 mL/min, and the detection wavelength was 395 nm.

### 3.4. Pharmaceutical Characteristics

The pharmaceutical properties were evaluated. The pH was measured 48 hours after preparation and after 3 months of storage and ranged from 7.2 to 7.5. Cream spreadability was determined by measuring the diameter of 0.5 g of cream between 2 plates under applied weights. The calculated area was used as a measure of spreadability. Quality-control procedures were conducted in accordance with the submitted protocol.

### 3.5. Microbial Quality Control

Microbial quality control of the CLA formulation was performed according to the British Pharmacopoeia. The cream sample was tested for total aerobic microbial count (TAMC), total yeast and mold count (TYMC), and the presence of specific pathogens, including *Staphylococcus aureus* and *Pseudomonas aeruginosa*. The pour-plate method was used for enumeration. Colony counts were performed after the specified incubation periods, and results were expressed as colony-forming units per gram (CFU/g) of product.

### 3.6. Sample Size

The sample size was calculated based on the primary clinical outcome (improvement rate) reported in a comparable study by Niazi et al. (12), which showed proportions of 0.30 in the intervention group and 0.05 in the control group. Using this difference in proportions, a 2-sided alpha of 0.05, and 80% power, a sample size of 32 participants per group was determined.

### 3.7. Patients' Eligibility

Eligible participants were older than 18 years, had a clinical diagnosis of mild-to-moderate CD, and had a TIS score between 1 and 8. All participants provided written informed consent.

Exclusion criteria were severe CD with TIS ≥ 9; corticosteroid or immunosuppressant use within the previous month or use of any dermatitis-related medication within the previous 2 weeks; pregnancy or lactation; history of childhood eczema; suspicious cases of CD; history of severe chronic infection; active cancer; cardiovascular or kidney disorders; asthma; alcohol addiction; scarring or hyperpigmentation at the lesion site; and glucose-6-phosphate dehydrogenase deficiency.

### 3.8. Randomization and Blinding

Seventy-three participants were screened, and 64 were enrolled and randomized. A statistician generated an allocation list using Random Allocation Software 2.0. Participants were assigned sequentially according to enrollment. The researchers, physicians, and statisticians remained blinded throughout the trial.

### 3.9. Intervention

Eligible participants were allocated to the intervention group (n = 32), which received CLA cream, or the control group (n = 32), which received base cream. Participants applied 1 fingertip unit (FTU) of cream to the affected area twice daily for 4 weeks. They were advised to wash the lesion with lukewarm water and a mild detergent. Participants were followed up at the end of weeks 2 and 4. At each visit, the affected skin areas were photographed and evaluated.

### 3.10. Outcome Measures

Biophysical skin properties, including transepidermal water loss (TEWL), sebum production, and stratum corneum hydration, were measured as primary outcomes under standardized conditions using the Cutometer® MPA 580 (EnviroDerm, UK). Assessments were conducted at baseline and after 2 and 4 weeks of intervention. The severity of CD was assessed using the Three-Item Severity Score (TIS), in which erythema, edema/papulation, and excoriations are each scored from 0 to 3 (13), at baseline and after 4 weeks. The severity of itching and discomfort was evaluated using a visual analog scale (VAS) (14).

As a secondary outcome measure, the Dermatology Life Quality Index (DLQI) questionnaire was used to assess patients' quality of life at baseline and at the end of the study (15, 16). DLQI scores range from 0 to 30, with higher scores indicating greater impairment (17).

### 3.11. Safety

Participants were instructed to report any adverse effects, with particular attention to redness, itching, and allergic reactions. Adverse events were documented and graded according to the Common Terminology Criteria for Adverse Events, version 5.0.

### 3.12. Statistical Analysis

Data were presented as mean ± standard deviation or as numbers. All analyses were performed using SPSS version 15 (SPSS Inc., Chicago, IL, USA). Repeated-measures analysis of variance with least significant difference post hoc tests, Friedman tests, and Wilcoxon tests were applied as appropriate. The Mann-Whitney test was used to compare outcomes between the 2 independent study groups.

Given the evaluation of six biophysical parameters as primary outcomes, a post hoc Bonferroni correction was applied to control the family-wise type I error rate. Accordingly, the adjusted significance threshold for these multiple comparisons was set at P < 0.008.

## 4. Results

### 4.1. Standardization of the CLA Formulation

The CLA formulation was standardized by quantifying total phenolic content using the Folin-Ciocalteu method, with gallic acid as the standard. The phenolic content was 2.15 ± 1.58 mg of gallic acid equivalent per gram of cream. All steps were repeated 3 times. In addition, the concentration of aristolochic acid in the *A. rotunda* extract was determined by HPLC and was 27.36 ppm (μg/g) in the extract.

### 4.2. Microbial Quality Control

The results demonstrated the absence of *S. aureus* and *P. aeruginosa* in 1 g of product. Both TAMC and TYMC were < 10 CFU/g, which is within the acceptable limits specified by the British Pharmacopoeia for topical preparations (TAMC ≤ 10^2^ CFU/g and TYMC ≤ 10^2^ CFU/g). These findings confirm that the cream complied with the required microbiological quality and safety standards.

### 4.3. Baseline Characteristics

**Figure 1.**
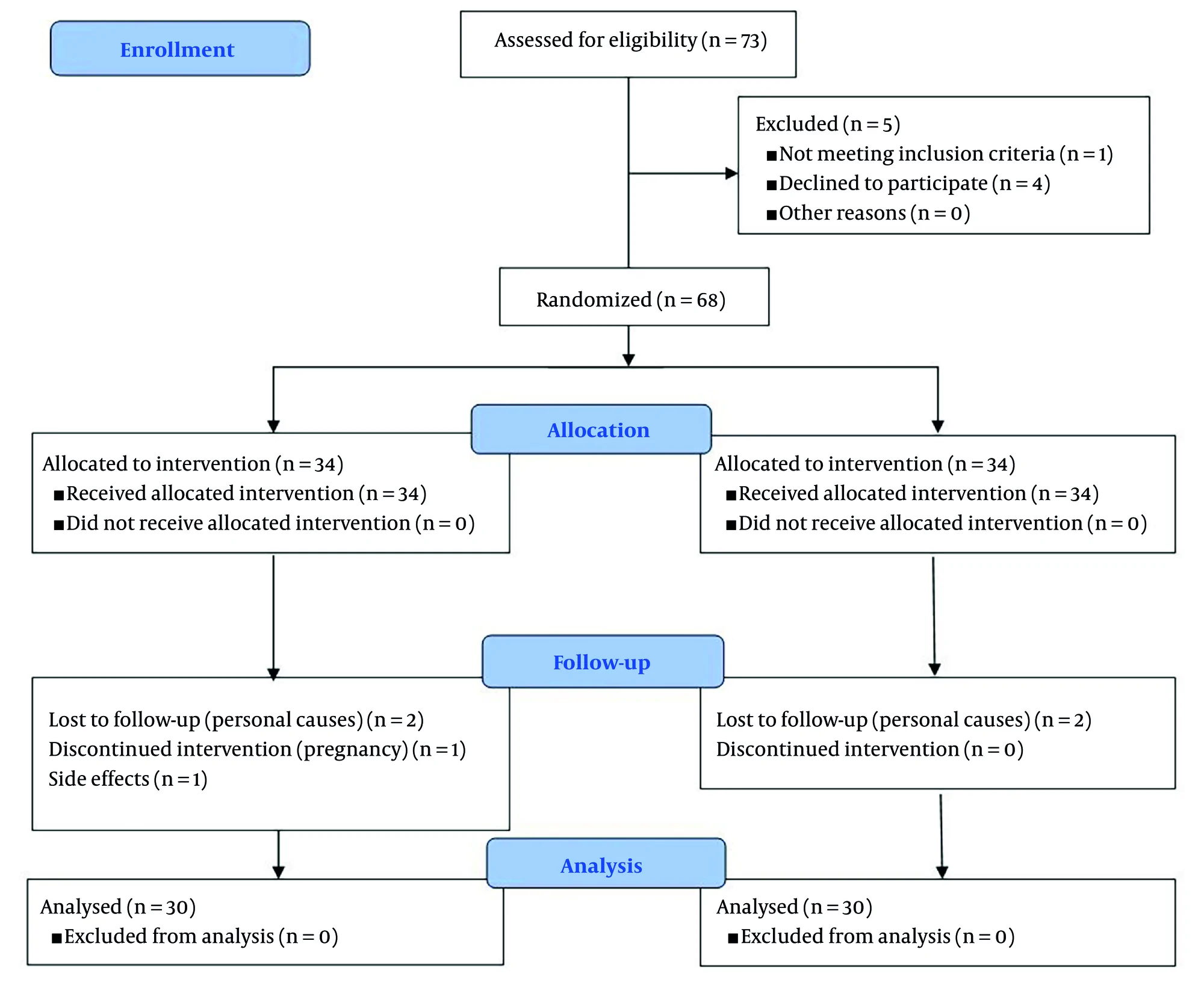
Detailed description of patient enrollment, randomization, and analysis (CONSORT diagram).

A total of 73 patients were assessed, and 64 were enrolled and allocated to the CLA or control group. Of these, 62 (96.8%) completed the trial and were included in the intention-to-treat analysis (ITT). Two participants discontinued the study in the CLA group (1 because of adverse effects and 1 because of pregnancy). Patient enrollment is shown in Figure 1.

Baseline demographic and clinical characteristics are shown in Table 1. No statistically significant differences were observed between the 2 groups in age, sex, weight, height, marital status, or occupational exposure.

**Table 1.** Demographic Data and Baseline Clinical Characteristics of Patients Participating in the Trial ^a^

Variables	CLA	Control	P Value
**Gender**			0.15 ^b^
Male	4	9	
Female	26	23	
**Mean age (y)**	41.10 ± 12.61	44.53 ± 11.33	0.26 ^c^
**Mean weight (kg)**	70.33 ± 16.05	71.48 ± 12.56	0.25 ^c^
**Mean height (m)**	1.64 ± 0.09	1.66 ± 0.09	0.75 ^c^
**Marital status**			0.86 ^b^
Single	10	10	
Married	20	22	
**Occupational exposure**			0.80 ^b^
Yes	16	18	
No	14	14	
**Disease duration (y)**	6.33 ± 8.29	6.34 ± 6.11	0.99 ^c^
**Disease zone**			-
Hands	24	21	
Feet	3	7	
Trunk	1	1	
Other	2	3	

^a^ Values are expressed as number or mean ± SD.

^b^ Chi-square test.

^c^ Independent-samples t test.

### 4.4. Clinical Assessments

In the within-group analysis of the CLA group after 4 weeks of intervention, TEWL significantly decreased from 35.97 to 28.32 (P = 0.008), while sebum production significantly increased from 6.75 to 19.65 (P = 0.006) (Table 2).

**Table 2.** Comparison of Skin Parameters Within Groups Before and After 2 and 4 Weeks of Intervention ^a^

Groups and Variables	Pretreatment	After 2 Weeks	Posttreatment	P-Value
**CLA group**				
TEWL	35.97 ± 17.07	29.99 ± 14.44	28.32 ± 13.20	0.008
Stratum corneum hydration	15.86 ± 21.48	27.05 ± 31.27	27.46 ± 31.27	< 0.001
Sebum	6.75 ± 11.19	18.73 ± 28.28	19.65 ± 21.89	0.006
pH	7.33 ± 1.46	7.00 ± 0.74	6.76 ± 0.48	0.161
**Control group**				
TEWL	28.897 ± 19.77	28.84 ± 14.93	32.44 ± 19.42	0.481
Stratum corneum hydration	11.35 ± 12.35	19.37 ± 16.07	20.77 ± 15.70	0.005
Sebum	19.90 ± 24.99	24.41 ± 36.19	26.75 ± 55.26	0.697
pH	7.16 ± 1.22	6.98 ± 1.09	6.88 ± 0.73	0.552

^a^ Values are expressed as mean ± SD.

**Figure 2.**
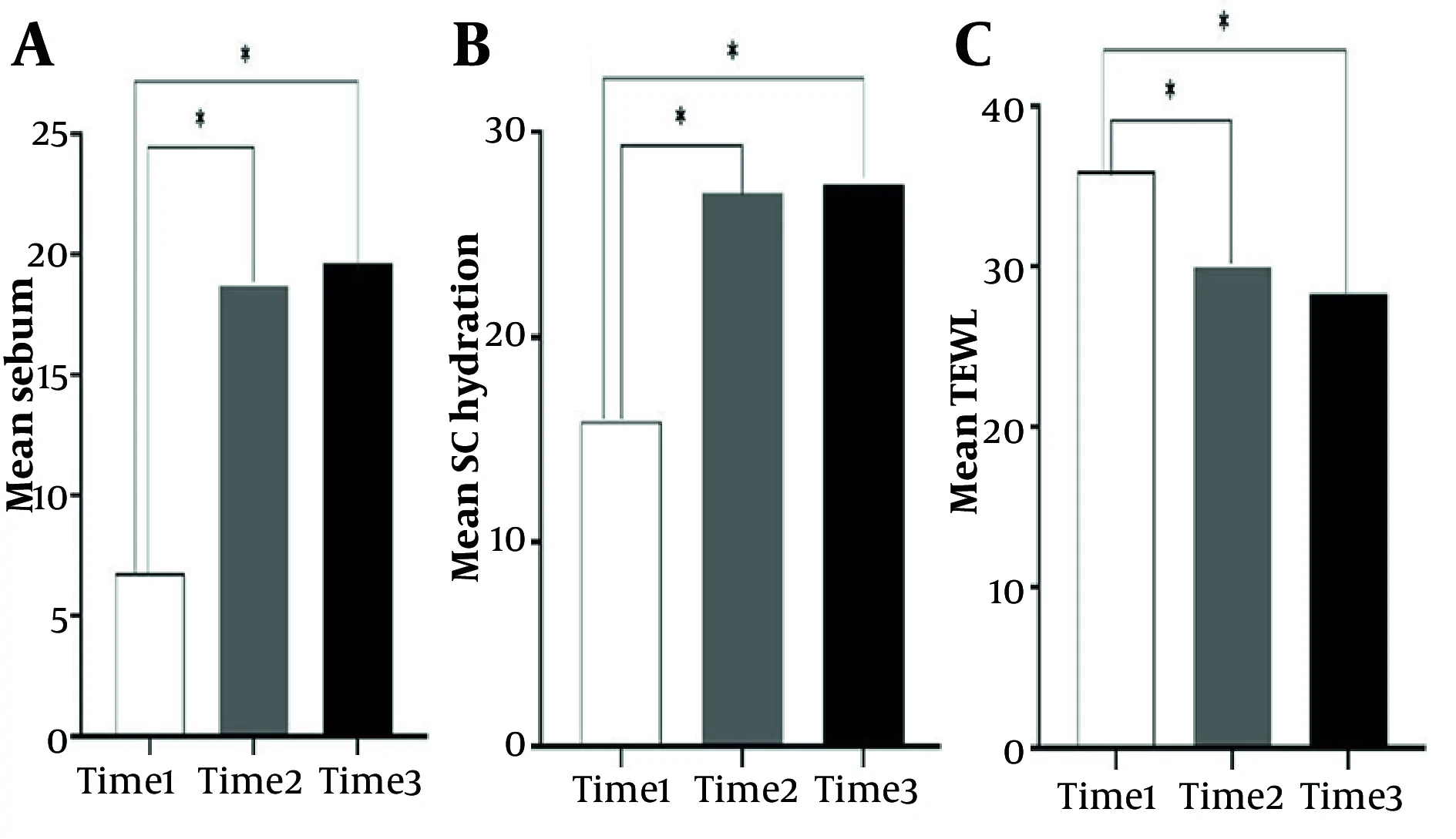
CLA-group sebum production, stratum corneum hydration, and TEWL after 2 and 4 weeks of intervention compared with baseline.

*Stratum corneum* hydration improved significantly in both the CLA and control groups, from 15.86 to 27.46 (P < 0.001) and from 11.35 to 20.77 (P = 0.005), respectively. The CLA group showed a numerically greater degree of recovery (Figure 2).

Between-group analysis showed significantly greater decreases in TEWL and TIS scores in the CLA group (P = 0.004 and P = 0.003, respectively) (Table 3).

**Table 3.** Improvement Rates of Parameters Between the 2 Groups After Treatment ^a^

Variables	CLA	Control	P-Value
**TIS**	3.10 ± 1.60	2.03 ± 1.46	0.003
**VAS**	4.00 ± 2.50	3.06 ± 2.55	0.150
**DLQI**	5.06 ± 5.50	2.71 ± 4.29	0.053
**TEWL**	7.64 ± 13.08	-3.54 ± 14.90	0.004
**Stratum corneum hydration**	-11.60 ± 14.13	-9.42 ± 11.99	0.515
**Sebum**	-12.90 ± 21.04	-6.84 ± 56.23	0.109

^a^ In contrast, the other parameters showed no statistically significant between-group differences (P > 0.008).

### 4.5. Safety

CLA was well tolerated, and no serious adverse effects were reported. In 1 participant, the lesions transformed into a papular form, and discontinuation of the cream was recommended.

## 5. Discussion

CD is a common disease that profoundly affects quality of life. Although immunosuppressive drugs are standard treatments, their long-term use is associated with adverse effects. Given these limitations and the global trend toward complementary and traditional therapies, further investigation in this field appears warranted (18). This trial evaluated, for the first time, the efficacy of CLA, a turmeric-based formulation from Persian medicine, in patients with mild-to-moderate CD. Application of CLA for 4 weeks resulted in a significant improvement in TEWL. A water gradient exists across the epidermis, causing passive diffusion of water from the inner layers toward the stratum corneum. This water loss from the skin, caused by evaporation in the absence of sweating, is termed TEWL (19). Disruption of the skin barrier and immune dysregulation can increase TEWL, leading to dry skin, pruritus, and impaired quality of life. Breaking this cycle is therefore important for clinical recovery. Decreased TEWL is positively associated with clinical improvement and better skin parameters (20). *Stratum corneum* hydration improved significantly within both groups; however, the between-group difference was not statistically significant (P = 0.515). The reduction in TIS scores was also notable (Table 3).

Curcumin, a polyphenolic, curcuminoid-rich component of *C. longa*, can inhibit the biosynthesis of prostaglandins and inflammatory mediators, including lipoxygenase, cyclooxygenase, tumor necrosis factor α, and interleukin 6 (21). In addition to its anti-inflammatory effects, curcumin has been shown to upregulate the expression of proliferating cell nuclear antigen in skin tissues, thereby promoting wound healing. This effect has been attributed to the stimulation of transforming growth factor β and its role in extracellular matrix production and angiogenesis. Because of these properties, *C. longa* is a candidate ingredient for moisturizers intended to repair the skin barrier (22).

Lawsone (2-hydroxy-1,4-naphthoquinone) is the main component of *L. inermis*. Although it has anti-inflammatory properties, it has also been identified as a cause of CD (23). Accordingly, the extraction process for *L. inermis* in this study was designed to minimize lawsone content while preserving antioxidant and free-radical-scavenging properties. The major constituents in the aqueous extract were ferulic acid, gallic acid, and luteolin (24). These compounds have demonstrated anti-inflammatory activity by decreasing tumor necrosis factor α and interleukin 6 and inhibiting nuclear factor κB activation (25). Aristolochic acid, one of the main constituents of *A. rotunda*, can regulate prostaglandin synthesis, inhibit phospholipase A, and decrease eicosanoids and platelet-activating factors (26).

The number of clinical trials and research articles on herbal treatments for CD remains small but has continued to increase. To our knowledge, no clinical trial has previously investigated this specific formulation. A related formulation is an ointment containing henna and curcumin (Alpha® Ointment). Studies have investigated Alpha ointment for wound healing and dermatitis and have reported positive results.

Three clinical trials conducted between 2001 and 2014 investigated the effects of *C. longa* on eczema. These studies reported wound-healing properties of *C. longa* treatments, attributed to enhanced angiogenesis and antimicrobial and anti-inflammatory effects. However, because most formulations contained combinations of herbal preparations, the evidence remains insufficient to establish the effect of *C. longa* alone in topical preparations (27). Supporting our findings, Suryawati et al. demonstrated that a *C. longa* nanoemulgel improved atopic dermatitis-like lesions in mice by repairing the skin barrier (28).

Few studies have investigated topical *L. inermis* for dermatitis. Our results are consistent with those of Niazi et al. (12), who found that topical *L. inermis* improved CD in lower-limb prosthesis users. Keshavarz et al. compared an *L. inermis* product with hydrocortisone for diaper dermatitis and found a better response in the *L. inermis* group (29).

No studies were found that investigated topical *A. rotunda* for dermatologic conditions. However, given its anti-inflammatory properties (30), this extract could plausibly be beneficial. The combination used in CLA cream may therefore produce a synergistic effect that contributes to the observed clinical effects.

### 5.1. Limitations

This study has several limitations. First, the sample size was relatively small. Second, because of the lack of a standard treatment for CD, comparison with an established reference therapy was not possible. Finally, a longer trial duration may have yielded more pronounced effects.

### 5.2. Conclusions

According to the study results, CLA cream was associated with reductions in TEWL and TIS scores. *Stratum corneum* hydration improved significantly within both groups but did not differ significantly between groups. CLA may therefore be considered a complementary remedy for improving symptoms in patients with mild-to-moderate CD.

## Data Availability

The dataset presented in the study is available on request from the corresponding author during submission or after publication. The data are not publicly available due to ethical considerations.

## Clinical Trial Registration Code:

IRCT20220321054336N1

https://irct.ir/search/result?query=IRCT20220321054336N1
